# Toll-like receptors: their roles in pathomechanisms of atopic dermatitis

**DOI:** 10.3389/fimmu.2023.1239244

**Published:** 2023-09-05

**Authors:** Risa Tamagawa-Mineoka

**Affiliations:** Department of Dermatology, Graduate School of Medical Science, Kyoto Prefectural University of Medicine, Kyoto, Japan

**Keywords:** atopic dermatitis, barrier, inflammation, innate immunity, stratum corneum, skin, toll-like receptors

## Abstract

The skin functions as a physical barrier and represents the first line of the innate immune system. There is increasing evidence that toll-like receptors (TLRs) are involved in the pathomechanisms of not only infectious diseases, but also non-infectious inflammatory diseases. Interestingly, it has been demonstrated that TLRs recognize both exogenous threats, e.g. bacteria and viruses, and endogenous danger signals related to inflammation, cell necrosis, or tissue damage. Atopic dermatitis (AD) is a chronic relapsing inflammatory skin disease, which is associated with impaired skin barrier function, increased skin irritability to non-specific stimuli, and percutaneous sensitization. The impairment of skin barrier function in AD allows various stimuli, such as potential allergens and pathogens, to penetrate the skin and activate the innate immune system, including TLR signaling, which can lead to the development of adaptive immune reactions. In this review, I summarize the current understanding of the roles of TLR signaling in the pathogenesis of AD, with special emphasis on skin barrier function and inflammation.

## 1 Introduction

**Figure 1:**
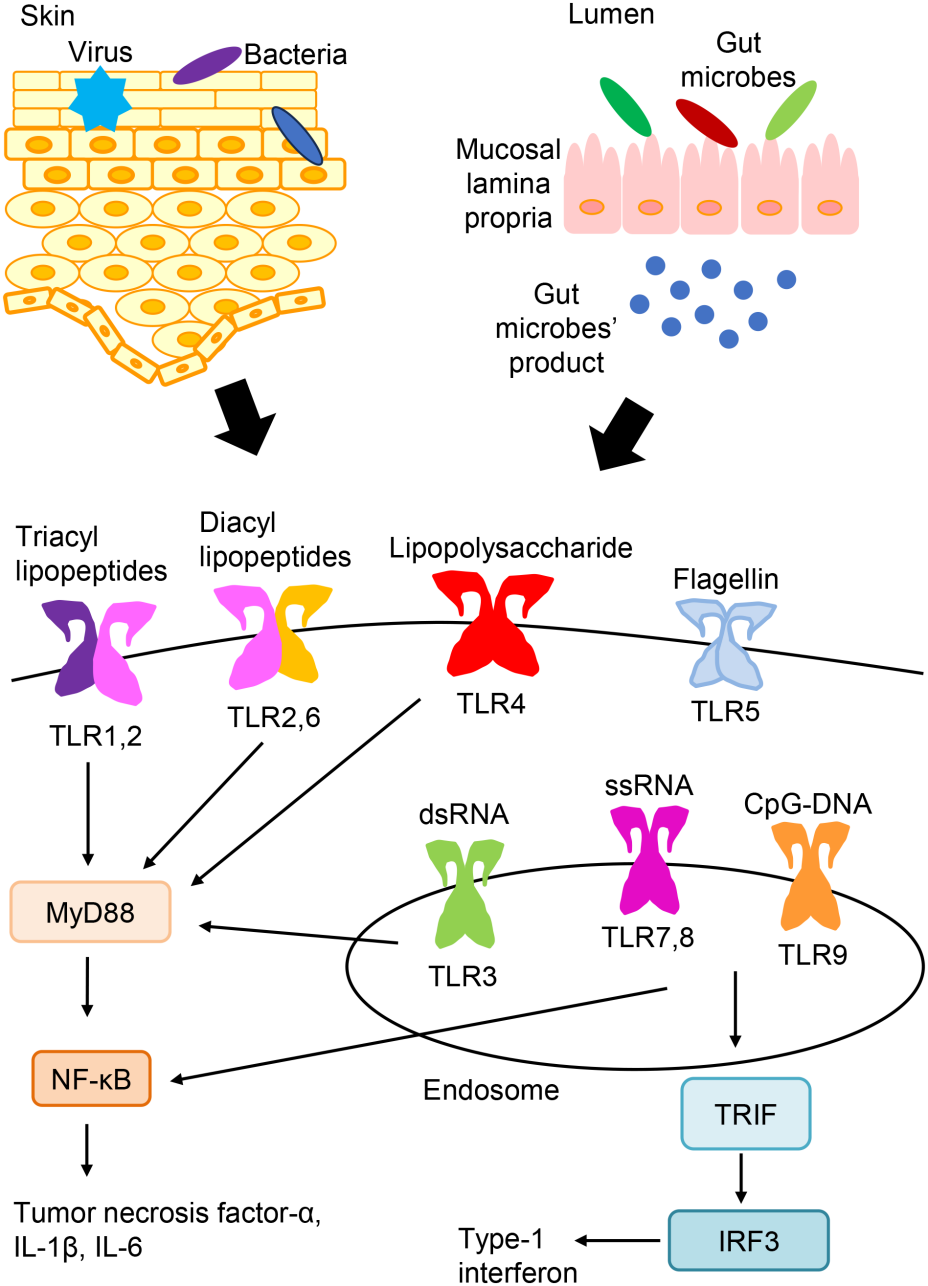
TLR expression and signaling. TLRs express on the cell surface (TLR1, TLR2, TLR4, TLR5, and TLR6) or in endosomes (TLR3, TLR7, TLR8, and TLR9). TLR ligands from bacteria, viruses, and so on are recognized and stimulate TLR signaling pathways. And, TLR signaling leads to the activation of NF-kB, interferon-regulatory factor, and their target genes, which induces the production of various antimicrobial and proinflammatory cytokines.

With respect to pathogen recognition by the innate immune system, characteristic molecular patterns that are exhibited by bacteria or viruses are recognized by pathogen sensors, which are termed pattern recognition receptors (PRRs) (1–3). The primary PRRs include toll-like receptors (TLRs), nucleotide-binding oligomerization domain-like receptors, and retinoic acid-inducible gene-I-like receptors. Regarding TLRs, ten kinds of TLRs have been found, and they are expressed on a variety of cells in humans (**Figure 1**) (1–3). TLR signaling can induce the activation of nuclear factor-kB (NF-kB), interferon-regulatory factor, and their target genes, resulting in the release of numerous antimicrobial and proinflammatory mediators (1–3).

Atopic dermatitis (AD) is a common chronic inflammatory skin disorder, affecting 2–20% of the general population, although its frequency varies with age and ethnicity (4, 5). The major manifestations of AD include dry skin, intense pruritus, and recurrent eczematous lesions. The pathomechanisms of AD are associated with impaired skin barrier function, enhanced skin irritability in response to non-specific stimuli, and percutaneous sensitization (4). In AD, a variety of complicating factors, e.g., irritants, house dust mites, pollen, food, microbial organisms, sweat, contact allergens, and scratching, can induce the development of skin inflammation (6). Recently, the innate immune system, including TLR signaling, has been shown to be involved in the pathomechanisms of AD. In this review, I summarize the roles of TLRs in cutaneous barrier function and inflammation in AD.

## 2 Stimulation of TLR signaling

### 2.1 TLR expressions in the skin

The skin consists of the epidermis, dermis, and subcutaneous layers. Various cell types express TLRs in the skin. In the epidermis, keratinocytes express TLRs 1-6, 9, and 10, and Langerhans cells express TLRs 1-6, 8, and 10 (7). Melanocytes express TLRs 2-4, 7, and 9 (7). In the dermis, resident cells, including mast cells (TLRs 1-7 and 9) and fibroblasts (TLRs 1-10), also express TLRs (7).

### 2.2 Exogenous ligands

TLRs recognize the common pathogen-derived molecular patterns shown by bacteria or viruses, which are termed pathogen-associated molecular patterns (3). The recognition of triacyl and diacyl lipopeptides requires the dimerization of TLR2 with TLR1 and TLR6, respectively. TLR3 detects double-stranded RNAs (dsRNAs). TLR4 recognizes bacterial lipopolysaccharides. TLR5 recognizes flagellin that makes up the flagella of bacteria. Both TLR7 and TLR8 detect single-stranded RNAs. TLR9 detects non-methylated cytosine-phosphate-guanine (CpG) DNA. TLRs express on the cell surface (TLR1, TLR2, TLR4, TLR5, and TLR6) or in endosomes (TLR3, TLR7, TLR8, and TLR9) (3).

Microbes, including bacteria, fungi, and viruses, are found on the skin surface and constitute a population that is peculiar to the skin, which is called the indigenous skin microbial flora (8). Such microbes can stimulate TLR signaling and are associated with cutaneous immune and inflammatory responses (**Figure 1**). Common skin commensal bacteria include *Propionibacterium acnes*, *Staphylococcus epidermitis* (*S. epidermitis*), and *S. aureus* (8). Bacterial colonization by *S. aureus* is the most common skin infection in AD patients (8). *S. aureus* is sensed by several PRRs, including TLR2 and TLR4 (1, 2). By acting on TLR2, *Propionibacterium acnes* may induce the cytokine release from follicular keratinocytes and macrophages, leading to inflammation (9). It has been reported that *Adam17* ^fl/fl^ *Sox9*-^Cre^ mice, an animal model ADAM17-deficiency in human, developed AD-like skin lesions with naturally occurring dysbiosis (10). *Corynebacterium mastitidis*, *S. aureus*, and *Corynebacterium bovis* sequentially appeared in the skin lesions. Furthermore, antibiotic specific for these bacterial species reversed dysbiosis and improved the skin lesions. These findings suggest that skin dysbiosis in the microbiome is related to the pathomechanisms of AD.

Demodex mites live in hair follicles or sebaceous glands (11). Demodex mites occur in all human ethnic groups, and they infect about 100% of the population up to old age (11). It was reported that the density of Demodex mites was increased in rosacea skin (11, 12). Sebum with an abnormal composition may induce the proliferation of Demodex mites in rosacea skin, leading to skin inflammation (11, 12). Furthermore, chitin, a polysaccharide present in the exoskeletons of insects, is known to stimulate TLR2 on keratinocytes (13).

Malassezia also exists in the human skin and is thought to be etiological factors for various skin disorders, including AD (6). Park et al. identified viral dsRNA segments in several clinical isolates of Malassezia species and revealed that the viral nucleic acid induced a TLR3-dependent immune response of dendritic cells (13). These findings suggest that a viral element included in Malassezia may be associated with the development of skin inflammation via TLR3 signaling.

In addition to the skin microbe, the gut microbiota is closely involved in the pathomechanisms of AD (14, 15). The gut microbiota and their products can be recognized by TLRs (**Figure 1**) (14). In the patients with AD, there are changes in the gut microbiota. While the good microbes including *Bifidobacterium* and *Lactobacillus* are reduced in AD patients (16, 17), there are higher proportions of *Clostridium*, *Staphylococcus* and so on in AD patients than those in non-allergic individuals (16, 18). Zachariassen et al. showed that a high and a low responding phenotype of AD, e.g. dermatitis and increased cytokine expressions, can be transferred with the gut microbiota to germ-free mice (19).

### 2.3 Endogenous ligands

Endogenous ligands stimulate TLRs and induce inflammation in non-infectious diseases (1). Many of the endogenous ligands are known to be released as a result of inflammation, cell necrosis, or tissue damage (1). Chaperones, such as heat shock protein and high mobility group box 1 (HMGB1) nuclear protein, are released from dead cells, and hyaluronic acid and biglycan are released from the extracellular matrix during inflammation (20). Moreover, TLR stimulation by low-density lipoprotein, fatty acids, or ω-2-carboxyethyl pyrrole was reported to be associated with dyslipidemia or oxidative stress (21). Such endogenous ligands act on TLR2 and TLR4, which function on the cell surface (1). In addition, TLR3, TLR7, TLR8, and TLR9 respond to self-derived nucleic acids (1).

## 3 Impaired skin barrier function in AD

### 3.1 Skin barrier function

The epidermis, which is the outermost layer of the skin, has important functions in the maintenance of skin barrier function (22–24). It consists of the stratum corneum, and granular, prickle, and basal cells. The stratum corneum contains three factors that are important for skin barrier function, sebum, intercellular lipids, and the natural moisturizing factor (22). Sebum derives from fat secreted by the sebaceous glands. A mixture of sebum and sweat covers the skin surface, preventing water transpiration by forming a sebum barrier. Intercellular lipids are lipids, e.g., ceramide and fatty acids, that fill the gaps between corneocytes (23). They play a role in the adhesive bonding of corneocytes and help to retain water in the skin. The natural moisturizing factor, which is responsible for water retention, is the name given to amino acids and their metabolites in the horny layer (23). These three factors function to maintain the skin’s barrier function and retain skin moisture.

### 3.2 Decreased filaggrin levels in AD

The granular-layer cells contain keratohyalin granules, the main component of which is profilaggrin, a phosphorylated histidine-rich high-molecular-weight protein (22, 24). In the keratinization phase, profilaggrin is decomposed to filaggrin monomers in the horny layer (22, 24). These filaggrin monomers aggregate to form keratin fibers, contributing to horny-layer cytoskeleton formation and the maintenance of physical strength. In addition, they are decomposed into amino acids in the most superficial layer of the horny layer. These amino acids and their metabolites are collectively termed the natural moisturizing factor. These factors are involved in the moisturization of the horny layer. Several studies have reported that 15-50% of the AD patients in Europe have filaggrin gene mutations, although there are regional differences (25–27). Filaggrin gene mutations are considered to prevent the production of normal profilaggrin and impair the barrier function of the skin.

### 3.3 Increased protease levels in AD

Other molecules that are important for the skin barrier formation include tissue kallikrein, a serine protease (28). In humans, 15 types of tissue kallikrein have been reported. In keratinocytes, the expression levels of kallikrein 5 (trypsin-type serine protease) and kallikrein 7 (chymotrypsin-type serine protease) are particularly high (29). In normal skin, these kallikreins may promote dekeratinization of the horny layer, with cell adhesion molecules used as a substrate (30). Interestingly, the overexpression of several types of kallikrein has been detected in the lesion sites of AD patients (31). In human kallikrein 7 transgenic mice, the spontaneous onset of chronic dermatitis was observed (32). On the other hand, lympho-epithelial Kazal-type inhibitor (LEKTI) tightly controls the activity of serine proteases, such as kallikrein 5 and kallikrein 7, in the epidermis (33). In LEKTI-deficient mice, horny-layer barrier hypofunction and skin inflammation are induced after the promotion of filaggrin decomposition (34). LEKTI is known to be an important molecule for the skin barrier function, as demonstrated by the fact that mutations in the gene of SPINK5, which encodes LEKTI protein, resulting in Netherton syndrome (35). In patients with this condition, AD-like symptoms and high serum IgE levels are seen (36).

### 3.4 Impaired tight junctions in AD

Tight junctions are structures that have an essential role in maintaining the skin barrier function (22, 24). The granular layer on the inside of the horny layer consists of 3 layers of cells, and the cells in the 2nd layer adhere tightly to each other through tight junctions, preventing the migration of water, ions, or soluble proteins (24). Tight junctions are consisted of transmembrane proteins, e.g. occludin, claudins, junction adhesion molecule proteins, and cytosolic scaffold proteins, including zonula occludens (22). Claudin-1 expression is greatly decreased in the skin of AD patients compared with that in non-atopic subjects (37). In addition, an association between claudin-1 polymorphisms and susceptibility to AD has been shown (37). These findings suggest that tight junction impairment is associated with the skin barrier dysfunction seen in AD patients.

### 3.5 Role of TLR signaling in skin barrier function of AD

In the skin of patients with AD, a significant imbalance in the microbiome, involving marked *S. aureus* colonization, is seen (8). *S. aureus* is sensed by several PRRs, including TLR2 (38). *S. aureus*-derived peptidoglycan, a TLR2 agonist, upregulates the expression of the tight junction proteins claudin-1, claudin-23, occludin, and zonulae occludens-1 in human keratinocytes (38). In addition, a TLR2 agonist increases the recovery of barrier function in the skin wounded by tape-stripping (38). Furthermore, TLR2 deficiency delays the skin barrier recovery after tape-stripping (38). In patients with AD, epidermal TLR2 expression is lower than that in non-atopic individuals, and is inversely correlated with transepidermal water loss (38). Yuki et al. also reported that tight junction-related barrier function was increased by a TLR2 agonist and the mechanism was inhibited by the knockdown of TLR signaling adaptor protein MyD88 (39). With regard to filaggrin-related barrier function, removal of profilaggrin/filaggrin products through extracellular vesicles in keratinocytes was done to regulate intracellular free filaggrin monomer content and prevent premature cell death during stratification (40). Such phenomenon was enhanced via TLR2-mediated sensing of *S. aureus* (40). These findings suggest that TLR2 signaling is involved in upregulation of the skin barrier function.

TLR3 signaling is also associated with skin barrier formation (41). The stimulation of TLR3 enhances the expression of several important genes that are related to skin barrier formation, e.g. the genes of ATP-binding cassette subfamily A, member 12; glucocerebrosidase; acid sphingomyelinase; serine palmitoyltransferase; glucosylceramide synthase; and transglutaminase 1 (41). In addition, TLR3 activation increases lamellar bodies and keratohyalin granules of keratinocytes, including profilaggrin (41). Borkowski et al. showed that the deficiency of TLR3 delayed the recovery of skin barrier function following ultraviolet B exposure (42). These findings indicate that TLR3 signaling contributes to the recovery of skin barrier function after skin injuries.

With regard to kallikrein and LEKTI, TLR1/2, 3, 5, and 2/6 ligands was reported to induce the expression of LEKTI in cultured human keratinocytes (43). On the other hand, trypsin and chymotrypsin-like serine protease activity are upregulated in human keratinocytes after the stimulation with a TLR3 agonist (43). The mRNA expression levels of kallikrein 6, kallikrein 10, kallikrein 11, and kallikrein 13 are also increased by a TLR3 agonist (43). TLR signaling may regulate kallikrein and LEKTI expression in keratinocytes.

## 4 Skin inflammation in AD

### 4.1 Immune and inflammatory responses in AD

In AD, an impaired skin barrier allows non-specific stimuli and potential allergens and pathogens to penetrate the skin, which can then activate immune and inflammatory responses (4–6). Recent studies have demonstrated that epithelial cell-derived cytokines, such as thymic stromal lymphopoietin (TSLP), interleukin (IL)-33, and IL-25 act on immune cells, including type-2 innate lymphoid cells (ILC2); induce type 2 immunity; and are deeply involved in the pathogenesis of AD (4, 5). Furthermore, not only type 2 helper T cells (Th2 cells), but also Th17 cells, Th1 cells, and Th22 cells have been reported to be involved in tissue remodeling, which is observed in the chronic phase of AD. Cytokines, such as IL-31 and TSLP, are also involved in itching, which can initiate a vicious cycle of inflammation (5).

### 4.2 Roles of TLR1/TLR2 in inflammation in AD

Single nucleotide polymorphisms in TLR1 have been suggested to contribute to susceptibility to AD (44). House dust mite allergens can be a complicating factor in AD. Jang et al. reported that upregulated mRNA and protein expression of TLR1, TLR6, IL-25, and IL-33 were detected in human AD skin lesions that had been subjected to high house dust mite sensitization (45). In addition, house dust mite extract upregulates the expression of TLR1, TLR6, IL-25, and IL-33 in cultured human keratinocytes (45). Furthermore, the knockdown of TLR1 inhibits the release of IL-25 and IL-33 (45). These findings suggest that house dust mite allergen-induced activation of TLR1 may induce polarization toward a type 2 immune response via the release of IL-25 and IL-33.

### 4.3 Role of TLR2/TLR6 in inflammation in AD

It is considered that microbial organisms, including *S. aureus*, may contribute to the pathomechanisms of AD (8). Iwamoto et al. reported that the expression level of TLR2, which senses *S. aureus*, on Langerhans cells in AD skin lesions was lower than that seen in healthy skin (46). Stimulation of Langerhans cells from normal skin with a TLR2 ligand leads to the maturation and increase of migratory activity (46). On the other hand, Langerhans cells from AD skin are less responsive to the TLR2 ligand (46). These findings suggest that TLR2-mediated signals are impaired in Langerhans cells from AD skin.

In addition, the associations between TLRs and high-affinity IgE receptor (FcϵRI) polymorphisms have been reported (47). More severe skin conditions are observed in AD patients having TLR2 gene (*TLR2*) rs4696480 major homozygotes and the FcϵRI α-chain gene (*FCER1A*) rs2252226 minor allele than in those characterized by the remaining combined rs2252226 and rs4696480 genotypes (47). These findings imply the involvement of TLRs-FcϵRI interactions in the pathomechanisms of AD.

Yu et al. showed that the basal mRNA expression level of thymus and activation-regulated chemokine (TARC)/C-C motif chemokine ligand 17 (CCL17) was elevated in peripheral blood mononuclear cells (PBMCs) from AD patients compared with that in PBMCs from healthy controls, while the basal mRNA expression levels of CCL8 and monocyte chemotactic protein-4 (MCP-4)/CCL13 were decreased in patients with AD (48). After stimulation with TLR2 ligands, the mRNA expression levels of regulated on activation, normal T cell expressed and secreted (RANTES)/CCL5, MCP-2/CCL8, MCP-4/CCL13, pulmonary and activation-regulated chemokine (PARC)/CCL18, and macrophage-derived chemokine (MDC)/CCL22 were higher in PBMCs from AD patients than in those from healthy controls (48)). In addition, Jang et al. reported that TLR6 deficiency suppressed the house dust mite allergen-induced upregulation of IL-25 or IL-33 expression (48)). These findings imply that TLR2 and TLR6 may induce polarization toward the type-2 immune response via the release of IL-25 and IL-33.

### 4.4 Role of TLR3 in inflammation in AD

TLR3 is expressed in the keratinocytes of both AD patients s and healthy individual (7, 49). The TLR3 expression levels of stratum corneum were elevated in the affected skin of AD patients compared with those in the unaffected skin of AD patients or healthy controls (49). Interestingly, the TLR3 expression levels of stratum corneum were correlated with the total intensity score, erythema score, oozing/crusting score, edema/papule score, excoriation score, lichenification score, and xerosis score (49). In addition, the water content was inversely correlated with the TLR3 expression levels in the stratum corneum of AD patients (49). Moreover, TLR3 (50–52) and its transcription factor interferon regulatory factor (53), especially in epithelial cells, is deeply involved in the mechanisms of allergic inflammation via cytokine and chemokine release in several murine models, including AD models.

Carbonic anhydrases are the enzymes that reversibly hydrate carbon dioxide and produce bicarbonate and protons, leading to regulation of pH and osmotic balance (54). The carbonic anhydrase II mRNA and protein expressions were increased in human primary keratinocytes upon treatment with a TLR3 agonist or the type 2 cytokines IL-4 and IL-13, seen elevated in AD (54). These findings suggest that carbonic anhydrase II may be involved in TLR3-related pathways in AD.

TLR3 signaling is also associated with the mechanisms of itching (7, 55). In the periphery, skin-resident cells, including mast cells and keratinocytes, can release various mediators, including nerve growth factor (NGF) and cytokines, that cause itching sensations. Deficiency of TLR3 suppresses the expression of NGF and scratching behavior in dry skin (55). Furthermore, the TSLP derived from keratinocytes acts upon primary sensory neurons, leading to occurrence of itching (56). Because the stimulation of TLR3 causes the production of TSLP by epithelial cells (57), the TSLP released from keratinocytes may stimulate primary sensory neurons through TLR3 signaling in the lesions of AD. In the spinal cord, TLRs, including TLR3, TLR4, and TLR7, are expressed in sensory neurons in dorsal root and trigeminal ganglions (55, 58, 59). TLR3 is expressed in small primary sensory neurons of dorsal root ganglions. Deficiency of TLR3 inhibits the histamine-dependent and -independent itching (55). These findings suggest that TLR3 signaling is associated with occurrence of itching.

### 4.5 Role of TLR4 in inflammation in AD

Many AD patients produce IgE against house dust mites or ticks (6). Trompette et al. reported that a tick component, Derp2, enhanced lipopolysaccharide-related TLR4 signals as a TLR4 adaptor-resembling molecule (60). In addition, they reported that the transnasal administration of Derp2 and lipopolysaccharide to mice increased the total IgE level, inducing tracheal inflammation, and that no such IgE induction was seen in TLR4-deficient mice (60). In addition to a Th2 immune responses, Th22 cells infiltrate the skin lesions of AD and release IL-22 that mediates epidermal thickening (5). Yoon et al. reported that endogenous TLR4 ligands were induced by tape-stripping, and caused the release of IL-23 from keratinocytes. This cytokine polarized dendritic cells to drive an IL-22 response in CD4^+^ T cells (61). These findings indicate that TLR4-mediated innate immune signals induce or enhance allergic responses.

On the other hand, some reports have suggested that TLR4 can regulate the development of AD (62, 63). In neonates, the occurrence of AD was related to decreased IL-10 production via TLR4, implying that TLR4-mediated immunomodulation during early life might affect the onset of AD (62). Lin et al. reported the immune regulatory function of TLR4 signaling in AD murine model induced by repeated epicutaneous application of a hapten, TLR4-deficient mice showed more severe AD symptoms and higher expression levels of inflammatory cytokines than wild-type mice after hapten challenge (63). In addition, the skin expression of TSLP was increased in TLR4-deficient mice compared with that in wild-type mice (63). Furthermore, the migration of dendritic cells into draining lymph nodes was increased in TLR4-deficient mice following hapten challenge (63). Therefore, the role of TLR4 signaling on the immune reactions in adult AD patients might differ depending on the experimental conditions.

With regard to the gut microbe, a previous cohort study showed a significant multiplicative interaction between *E. coli* and TLR4 SNP rs10759932 in children (64). In addition, *E coli* colonization was related to a decreased risk of sensitization only in children with the rs10759932 TT genotype (64). Furthermore, West et al. reported that *Ruminococcaceae* in stool samples was decreased in the infants developing AD compared with that in healthy individuals and was inversely related to TLR2-induced IL-6 and tumor necrosis factor (TNF)-α. *Enterobacteriaceae* (a genus of Proteobacteria phylum) was negatively associated with TLR4-induced TNF-α (65). Taken together, these findings suggest that the gut microbe can affect the development of AD via TLR signaling in early life.

## 5 Conclusions

In conclusion, TLRs are expressed on various types of cells, including leukocytes and skin-resident cells. They detect not only exogenous threats, including bacteria and viruses, but also endogenous danger signals related to inflammation, cell necrosis, or tissue damage. In the skin of AD patients, several cell types can release numerous mediators via TLR signaling and are deeply involved in skin barrier formation and inflammation. These findings could lead to the establishment of novel TLR signaling-based treatments for AD based on the concept of innate immunity.

## Author contributions

RT-M wrote the article and approved the submission of the article.
